# Predictive Value of ^99m^Tc DPD Bone SPECT/CT Uptake Ratio for Culture Results in Lower Limb Osteomyelitis

**DOI:** 10.3390/diagnostics15162109

**Published:** 2025-08-21

**Authors:** Hyun Suk Shin, Min Bom Kim

**Affiliations:** Department of Orthopaedic Surgery, Seoul National University Hospital, College of Medicine, Seoul National University, Seoul 03080, Republic of Korea; carlshin@naver.com

**Keywords:** bone SPECT/CT scan, chronic osteomyelitis (COM), microbiological identification, pathogen detection, culture positivity

## Abstract

**Background/Objectives**: The diagnosis of osteomyelitis is typically based on clinical suspI icion supported by imaging and lab findings. Various nuclear medicine imaging, including bone SPECT/CT, is emerging as an effective tool to guide the diagnosis of osteomyelitis. This study investigates whether the preoperative ^99m^Tc DPD bone SPECT/CT uptake \ratio correlates with intraoperative tissue culture positivity in patients with suspected lower extremity osteomyelitis. **Methods**: We retrospectively reviewed 46 patients who underwent surgery for suspected osteomyelitis of the lower extremity between February 2020 and May 2025. Bone SPECT/CT was performed using ^99m^Tc DPD, and uptake values were measured using Syngo.via software. Lesion-to-Background Ratio (LBR) was calculated by comparing uptake in the lesion with the contralateral bone. Intraoperative culture was conducted at the region with high uptake in SPECT/CT. **Results**: Among the 46 patients who underwent surgery, 28 had positive tissue cultures, and 18 were negative. The mean LBR was significantly higher in culture-positive cases (14.5 ± 4.5) than in culture-negative cases (6.8 ± 8.0, *p* = 0.0002) Inflammatory markers (WBC, ANC, ESR, CRP) and the antibiotic-free interval before surgery did not significantly differ between groups or correlate with LBR. ROC analysis identified an LBR threshold of 9.44, yielding a sensitivity of 71.4% and specificity of 88.9% for predicting positive cultures (AUC = 0.81). **Conclusions**: ^99m^Tc DPD bone SPECT/CT uptake ratio may serve as a useful tool for the preoperative assessment of suspected lower extremity osteomyelitis, providing a more reliable prediction of intraoperative microbial culture results compared to serum inflammatory markers or the duration of antibiotic-free intervals. High tracer uptake may also be observed in various other conditions and thus should be interpreted in a multidisciplinary context in conjunction with other modalities.

## 1. Introduction

Osteomyelitis, which encompasses both chronic and post-traumatic cases, represents a complicated bone and bone marrow infection that develops from hematogenous spread or occurs as a result of open fractures or surgical interventions with internal fixation devices [1,2,3]. Accurate diagnosis and management of osteomyelitis require identification of both the site of infection and the causative pathogens [4].

In addition to clinical history taking and physical examination, the diagnostic workup of osteomyelitis typically includes laboratory evaluation of inflammatory markers and imaging studies [4]. Serum inflammatory markers, such as erythrocyte sedimentation rate (ESR), C-reactive protein (CRP), and leukocyte count, are inexpensive, widely available, and useful for monitoring treatment response [5]. However, their diagnostic specificity is limited, as elevations may occur in various infectious or inflammatory conditions unrelated to the bone [6]. Furthermore, they do not provide information on the precise anatomical location of infection, making them insufficient as standalone diagnostic tools.

The current imaging methods which detect infectious bone lesions in osteomyelitis present multiple difficulties for diagnosis [7]. Radiography, CT, MRI, FDG-PET, and SPECT have been employed for diagnosing osteomyelitis, according to previous research. CT provides detailed structural information but lacks functional assessment. MRI serves as the primary diagnostic tool because of its excellent sensitivity and specificity, but it faces challenges when used in postoperative cases with hardware and when edema leads to overestimated infection spread [8].

There is growing interest in using nuclear medicine imaging in the diagnosis and management of osteomyelitis. These imaging modalities can help identify multifocal lesions and complement traditional imaging. However, the ability of planar bone scintigraphy to pinpoint infection foci and support surgical planning remains limited [9]. Recent studies have emphasized the diagnostic performance of other nuclear imaging modalities. PET/CT and SPECT/CT combine metabolic imaging with precise anatomical localization, enabling accurate identification of infection sites even in the presence of orthopedic hardware. ^18^F-FDG PET showed effectiveness in diagnosing and localizing osteomyelitis [10,11,12,13] and demonstrated high sensitivity even in the presence of antibiotic use [14,15].

Bone SPECT/CT has demonstrated high accuracy in the evaluation of osteomyelitis [16,17]. The pathophysiology of osteomyelitis involves bacterial invasion of bone tissue, leading to an inflammatory cascade characterized by hyperemia, increased vascular permeability, and stimulation of osteoblastic activity [18]. Bone-seeking radiotracers, such as ^99m^Tc-labeled phosphonates, localize to areas of active bone turnover and increased blood flow, enabling bone scintigraphy to visualize active infection [19]. However, tracer uptake is not entirely specific and can also occur in non-infectious conditions, including recent fractures, postoperative changes, degenerative joint disease, inflammatory arthritis, and certain neoplastic processes, which may result in false-positive findings [16]. Still, several studies have evaluated osteomyelitis with SPECT. For example, studies in patients with diabetic foot osteomyelitis have shown that its diagnostic performance is comparable to that of MRI, which is considered the reference standard for this condition [20]. It has also been applied in oral and maxillofacial surgery to assist in determining resection boundaries for osteomyelitis in skull and facial regions [21,22].

Quantitative and semiquantitative approaches using bone SPECT/CT offer an objective and reproducible means of interpreting data [23]. These methods provide standardized thresholds and allow longitudinal comparison of tracer uptake, which enables assessment of disease progression or treatment response over time [24]. Recently, such approaches have been applied to mandibular osteomyelitis, comparing uptake values over time to assess treatment response [25]. Quantitative ^67^Ga SPECT/CT analysis has shown better sensitivity and specificity of diagnosing lower limb osteomyelitis compared to qualitative analysis [26].

Despite these developments, few studies have evaluated the use of bone SPECT/CT for surgical planning or predicting causative organisms in extremity osteomyelitis [21]. Building upon our previous work which investigated the utility of ^99m^Tc DPD bone SPECT/CT in localizing osteomyelitis for surgical debridement [27], this study aims to determine the relationship between bone SPECT/CT uptake ratio and intraoperative culture results in suspected lower extremity osteomyelitis.

## 2. Materials and Methods

### 2.1. Patient Selection

This retrospective study reviewed patients who underwent bone ^99m^Tc DPD SPECT/CT at the orthopedic department of our institution between February 2020 and June 2025 for suspected lower extremity osteomyelitis. The parallel arrangement of long bones in lower limbs enabled a reliable comparison of images, so only lower limb cases were included.

In our clinical setting, osteomyelitis was suspected in patients presenting with one or more clinical signs suggestive of bone infection (e.g., localized pain, swelling, erythema, or purulent drainage), particularly when accompanied by abnormal preliminary imaging or elevated inflammatory markers. For fracture-related cases, suspicion was further guided by the recently proposed international consensus definition for fracture-related infection (FRI) [3]. Patients presenting with findings suggestive of osteomyelitis underwent SPECT/CT to assess bone involvement.

Patients were categorized into two clinical management pathways depending on SPECT/CT findings. Those without marked radiotracer uptake were presumed not to have deep bone infection and were managed conservatively with oral antibiotics and local wound care or underwent soft tissue procedures if required. Patients with marked bone uptake or other definitive signs of infection underwent surgical management, including debridement of the infected bone, removal or replacement of internal fixation hardware, and soft tissue reconstruction. Intraoperative tissue specimens were obtained from the region showing the highest uptake on SPECT/CT, later referred to as the volume of interest (VOI).

**Figure 1:**
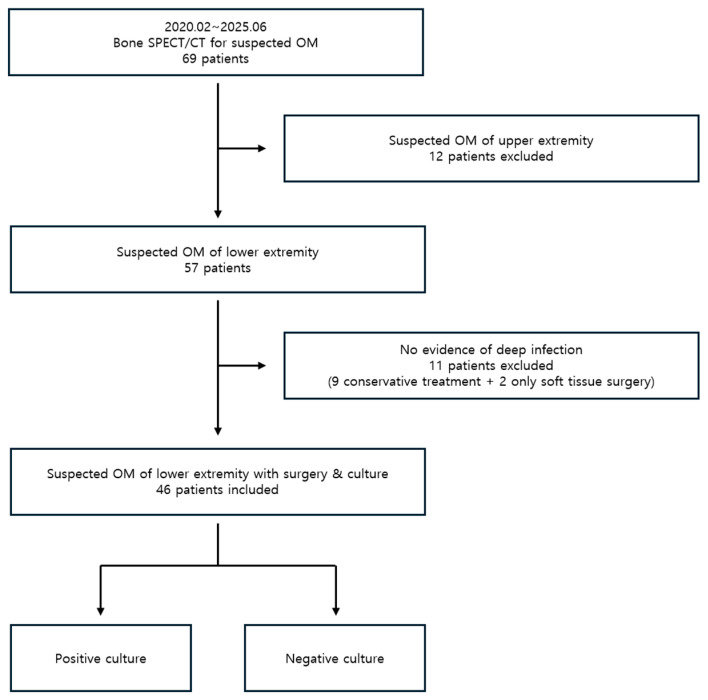
Flowchart of patient selection and inclusion process.

A total of 69 patients underwent ^99m^Tc DPD bone SPECT/CT for suspected osteomyelitis during the study period. Of these, 12 patients with suspected upper extremity osteomyelitis were excluded. Among the remaining 57 patients with lower extremity involvement, 11 showed no evidence of deep infection on SPECT/CT and were excluded from the analysis, as this study focused on bone-related infections. Of these, nine patients with no clear evidence of infection were treated conservatively, and two patients with only superficial involvement underwent soft tissue surgery. The remaining 46 patients underwent surgical treatment and intraoperative tissue culture based on bone SPECT/CT findings suggestive of infection. Therefore, the final 46 patients were enrolled in the study (Figure 1).

### 2.2. SPECT/CT Imaging Protocol and Quantification

Preoperative bone SPECT/CT was performed within one month prior to surgery, following intravenous injection of 0.74 to 1.1 GBq of ^99m^Tc DPD. Imaging was conducted 2–4 h post-injection using a hybrid SPECT/CT system (Discovery NM/CT 670; GE Healthcare, Milwaukee, WI, USA) equipped with dual-head gamma cameras using sodium iodide crystal detectors and low-energy high-resolution parallel-hole collimators. SPECT acquisition parameters included a 360° rotation, 60 projections (30 per head), step-and-shoot mode, 20 s per projection, a 128 × 128 matrix, and a 15% energy window centered at 140 keV. CT was performed using an integrated 16-slice BrightSpeed system with Care Dose automatic exposure control, 120 kVp, and 2.5–3.0 mm slice thickness. SPECT data were reconstructed using ordered-subset expectation maximization (OSEM) with scatter and CT-based attenuation correction and fused with CT images for anatomical localization. Regions of high tracer uptake were color-coded during initial interpretation by nuclear medicine physicians to facilitate visual identification of potential infection foci and to guide precise placement of the volume of interest (VOI) for quantitative analysis.

For uptake ratio analysis, DICOM images were exported to Syngo.via software (version VB80A; Siemens Healthcare, Erlangen, Germany). A spherical volume of interest (VOI) of approximately 10 cm^3^ was manually placed over the lesion showing the highest uptake, and a corresponding VOI was placed in the anatomically equivalent site on the contralateral limb. Voxel values were expressed as counts per voxel, which normalizes for VOI size and enables reproducible semiquantitative comparison across patients. Lesion-to-Background Ratio (LBR) was calculated as the ratio of the maximum uptake in the lesion VOI to the mean uptake in the contralateral VOI, a commonly used scheme that captures peak lesional activity while providing a stable background reference and reducing inter-patient variability through side-to-side normalization. This max/mean approach has been applied in previous studies for quantification in both bone SPECT/CT and PET/CT.

### 2.3. Preoperative Laboratory Testing

Complete blood counts and inflammatory markers including white blood cell count (WBC), absolute neutrophil count (ANC), erythrocyte sedimentation rate (ESR), and C-reactive protein (CRP) were obtained a day before surgery.

### 2.4. Statistical Analysis

All statistical analyses were performed using Python version 3.7.0.0 (Python Software Foundation, Wilmington, DE, USA). Categorical variables were compared between the culture-positive and culture-negative groups using Fisher’s exact test or chi-square tests, and continuous variables were compared using two-sided *t*-tests. The Pearson correlation method was applied to assess linear associations between the Lesion-to-Background Ratio (LBR) and laboratory inflammatory markers (WBC, ANC, ESR, CRP), as well as preoperative antibiotic duration. Receiver operating characteristic (ROC) curve analysis was conducted to evaluate the predictive performance of LBR for intraoperative culture results. The area under the curve (AUC) was calculated, and the optimal cutoff value was determined using Youden’s index (maximum [sensitivity + specificity − 1]). Sensitivity and specificity at the optimal cutoff were then derived. Multivariable logistic regression analysis was performed to determine whether LBR was an independent predictor of culture positivity after adjusting for potential confounders. Propensity score matching was used to adjust for preoperative antibiotic duration (measured in days) between groups, assuming conditional independence (no unmeasured confounders) and sufficient overlap (common support) in covariate distributions. A *p*-value < 0.05 was considered statistically significant for all tests.

## 3. Results

### 3.1. Tissue Culture Outcomes

Among the 46 patients who underwent surgery, intraoperative tissue cultures yielded positive results in 28 cases (60.9%) and negative results in 18 cases (39.1%). The mean age of the culture-positive group was 58.3 ± 17.4 years, compared to 56.8 ± 16.9 years in the culture-negative group. No statistically significant differences were observed between the groups in terms of age or sex. The duration of antibiotic use within the four weeks prior to surgery was 6.7 ± 12.5 days in the positive group and 13.4 ± 10.7 days in the negative group. Although this difference did not reach statistical significance, the mean duration in the negative group was twice that of the positive group (Table 1).

There were no statistically significant differences in preoperative laboratory blood test results between the two groups.

The distribution of pathogens identified in the culture-positive group was as follows: Methicillin-resistant *Staphylococcus aureus* (MRSA) in nine cases, Methicillin-sensitive *Staphylococcus aureus* (MSSA) in eight cases, Methicillin-resistant *Staphylococcus epidermidis* (MRSE) in four cases, Pseudomonas aeruginosa in two cases, Escherichia coli in two cases, Streptococcus dysgalactiae, Corynebacterium striatum, Streptococcus sanguinus in one case each.

### 3.2. Bone SPECT/CT Imaging

**Figure 2:**
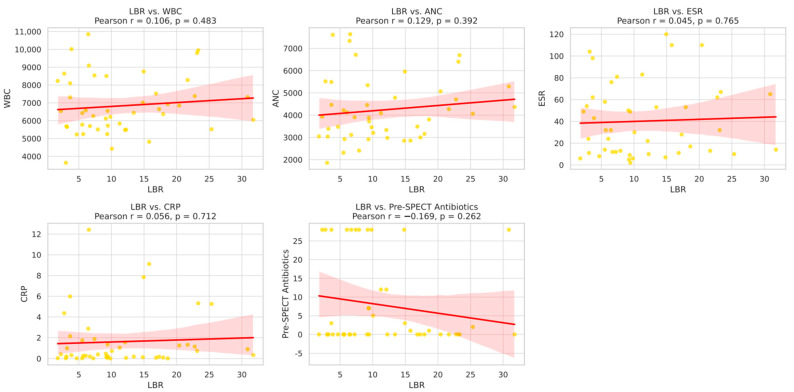
Correlation between LBR and inflammatory markers (WBC, ANC, ESR, CRP) and pre-SPECT antibiotics administration period within 4 weeks. (The yellow dots indicate individual patient data points, the red line indicates the regression line, and the red shaded area indicates the 95% confidence interval.)

The mean LBR value in the entire cohort was 11.5 ± 7.8. In the culture-positive group, the mean LBR was 14.5 ± 4.5, which was significantly higher than that of the culture-negative group (6.8 ± 8.0, *p* < 0.001). A linear correlation analysis was performed between the LBR values and preoperative blood test results. The Pearson correlation coefficients for WBC, ANC, ESR, and CRP were 0.106, 0.129, 0.045, and 0.056, respectively, all of which were below 0.3, indicating no meaningful correlation. The Pearson correlation coefficient between the LBR and the duration of antibiotic administration before SPECT/CT was 0.169, which also showed no statistical significance (Figure 2).

**Figure 3:**
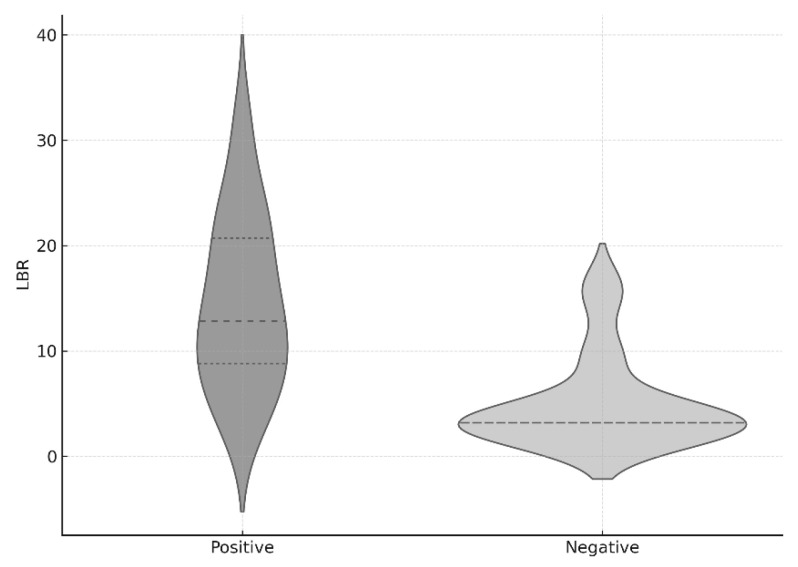
Distribution of LBR values after propensity score matching based on pre-SPECT antibiotic use.

Given that the pre-SPECT/CT antibiotics administration period might affect the statistical analysis of LBR between the two groups, propensity score matching was conducted to adjust for this variable. The results remained statistically significant after matching (*p* < 0.001) (Figure 3).

### 3.3. Microbiological Analysis

Among the culture-positive patients, eight cases were MSSA, nine cases were MRSA, and four cases were MRSE. Among the *Staphylococcus* species, 13 isolates were methicillin-resistant and 8 were methicillin-susceptible. The mean LBR values for the methicillin-resistant and methicillin-susceptible *Staphylococcus* groups were 15.8 ± 8.2 and 14.5 ± 9.8, respectively, with no statistically significant difference between the two groups (*p* = 0.754). However, the small sample size in the subgroup analysis limits the statistical power of this comparison. (see Discussion for details).

### 3.4. ROC Analysis

**Figure 4:**
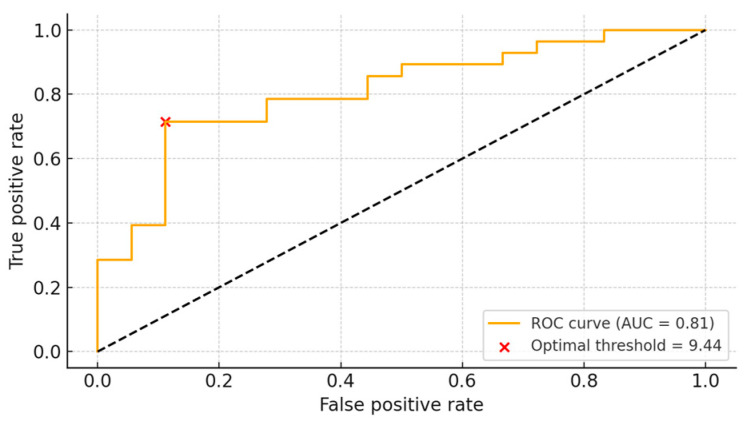
ROC curve for LBR values.

We evaluated how sensitivity and specificity varied according to different LBR thresholds. The optimal cutoff value was determined to be 9.44. At this threshold, the sensitivity was 71.4%, and the specificity was 88.9%, indicating that LBR could reasonably predict the likelihood of a positive bone culture. The area under the curve (AUC) was calculated to be 0.81 (Figure 4).

### 3.5. Multivariable Logistic Regression Analysis

Multivariable logistic regression was performed for two variables, LBR and preoperative antibiotics use within four weeks. The odds ratio for LBR was 1.29 (95% CI [1.13–1.47], *p*-value < 0.001) and the odds ratio for preoperative antibiotics use was 1.01 (95% CI [0.95–1.09], *p*-value 0.678). The final multivariable logistic regression model is presented below.

(1) $$Logit\;\left(p\right)=-2.263+0.254\times \left(LBR\right)+0.014\times (Preop\;antibiotics\;use\;within\;4\;weeks)$$

### 3.6. Case

A 45-year-old man sustained an injury while riding a motorcycle and was diagnosed with closed mid-shaft fractures of the left tibia and fibula. At another hospital, he underwent open reduction and internal fixation. The tibia was fixed using a metal plate and screws, while the fibula was treated with intramedullary fixation.

Starting five months after the surgery, pus began to discharge from the wound. At 8, 12, and 14 months postoperatively, the patient underwent a total of three procedures at the same hospital, including removal of the tibial implant and debridement. Tissue cultures were performed during all three surgeries: one was negative, and two yielded methicillin-sensitive *Staphylococcus aureus* (MSSA).

**Figure 5:**
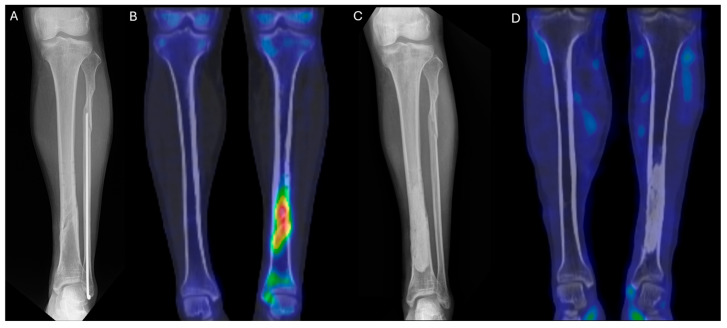
(**A**) Preoperative lower leg AP X-ray. (**B**) Preoperative ^99m^Tc DPD bone SPECT/CT coronal image. (**C**) Postoperative lower leg AP X-ray at 1 month. (**D**) Postoperative ^99m^Tc DPD bone SPECT/CT coronal image at 6 months, showing no hypermetabolic uptake, consistent with infection control.

**Figure 6:**
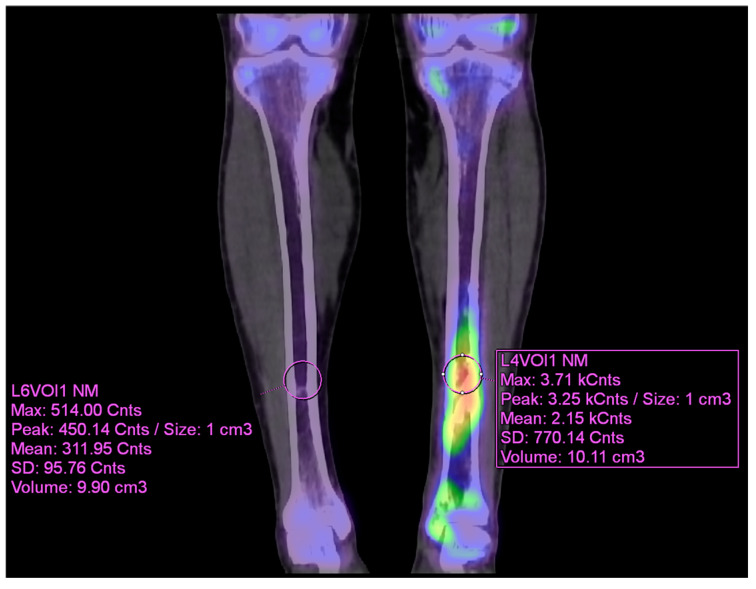
VOI (volume of interest) analysis of preoperative ^99m^Tc DPD bone SPECT/CT coronal image (contralateral side and affected side) using Syngo.via.

As the infection did not improve, the patient visited our outpatient clinic. He had been receiving intravenous and oral ciprofloxacin for 10 months since the 8-month postoperative day. After the initial visit to our clinic, ^99m^Tc DPD bone SPECT/CT scan was performed, revealing areas of increased uptake, as shown in the images below (Figure 5 and Figure 6).

The patient then underwent surgery, during which extensive debridement was performed on the suspected infected intramedullary lesion. Tissue culture was also taken from the site with the highest uptake on the SPECT/CT scan. Some intramedullary bone marrow was also removed. Antibiotic-impregnated bone cement was inserted into the debrided area.

After surgery, the patient received intravenous antibiotics for two weeks during hospitalization and continued with oral antibiotics for three weeks as an outpatient. At one month postoperatively, plain radiographs showed no specific findings, and at six months, a follow-up ^99m^Tc DPD bone SPECT/CT scan confirmed the absence of high-uptake lesions, indicating that the infection had been successfully controlled. The patient remained free of recurrence and infection for two years.

## 4. Discussion

This is the first study to retrospectively compare the ratio of radiotracer uptake in bone SPECT/CT between the osteomyelitis lesion and the contralateral limb in culture-positive and culture-negative groups. The LBR (Lesion-to-Background Ratio) values showed a statistically significant difference. The significantly higher LBR observed in culture-positive cases likely reflects more pronounced radiotracer accumulation in clinically active infections, whereas culture-negative cases may have included patients with reduced metabolic activity, due to prior antibiotic exposure or non-infectious conditions, leading to lower or more variable uptake values. Although there was no statistically significant difference in the duration of preoperative antibiotic use within the four weeks prior to surgery, the average duration differed by two-fold (13.4 vs. 6.7 days), suggesting that preoperative antibiotic use may influence the yield of intraoperative cultures.

To determine if antibiotic duration influenced LBR, we performed a propensity score matching analysis. The results from the analysis confirmed that LBR demonstrated statistical significance which means that its results are independent of preoperative antibiotic administration. Multivariable logistic regression repeatedly confirmed that LBR remained an independent predictor of culture positivity after adjusting for potential confounders. The analysis of LBR for clinical application using ROC curve methods established an optimal threshold at 9.44. An LBR value exceeding 9.44 enabled positive culture detection with a 71.4% sensitivity rate and an 88.9% specificity rate. The prediction accuracy of LBR for culture results reached 81% as shown by the area under the curve (AUC) which measured 0.81. This suggests that the radiotracer uptake ratio between the lesion and the contralateral limb may serve as a potentially meaningful indicator for distinguishing between culture-positive and culture-negative cases.

Preoperative blood test outcomes between culture-positive and culture-negative patients did not reveal distinguishable results using standard laboratory markers such as WBC, ANC, ESR and CRP. The linear regression analysis confirmed that LBR cannot be predicted from blood markers because the values showed no correlation with WBC, ANC, ESR or CRP. The absence of significant correlations between LBR and systemic inflammatory markers (WBC, ANC, ESR, CRP) may be explained by the fact that LBR reflects localized bone metabolic activity, whereas these laboratory parameters reflect systemic inflammatory response. In chronic or localized osteomyelitis, systemic markers may remain within normal limits despite active infection, particularly in patients with prior antibiotic exposure or underlying comorbidities, thereby attenuating the correlation.

ROC analysis was also performed for the four-week preoperative antibiotic discontinuation period. The AUC was 0.69 with a threshold value of four, yielding a sensitivity of 71% and specificity of 65%. The literature on whether preoperative antibiotic use affects intraoperative culture results is inconsistent. Some studies suggest reduced detection with preoperative antibiotics, while others state that use up to seven days prior has no significant effect [28,29,30,31]. We also examined whether antibiotic resistance affects LBR by comparing cases of methicillin-resistant and methicillin-susceptible *Staphylococcus* species. No statistically significant difference in LBR values was observed between these groups, suggesting that antibiotic resistance does not influence LBR. Despite the relatively small sample size (*n* = 46), post hoc power analysis based on the observed LBR difference between culture-positive and -negative groups (Cohen’s d = 1.18) indicated a statistical power of 96.9% at α = 0.05. In contrast, the subgroup analysis comparing methicillin-resistant and methicillin-susceptible *Staphylococcus* species had a post hoc power of only 6.1% (Cohen’s d = 0.15), indicating that this analysis was markedly underpowered to detect small differences.

One study in 2020 investigated the predictive value of CT attenuation for pathogen detection in vertebral osteomyelitis. Unlike the present study, that analysis focused on spinal bones and used CT—not nuclear imaging—to compare attenuation values with adjacent vertebrae to infer culture results. That study also found CRP to be a useful predictor, whereas our study did not, which may be attributable to anatomical differences or to the unreported antibiotic exposure in their cohort [32]. Another similar study using ^18^F-FDG PET in chronic osteomyelitis was conducted in 2017 [33]. That study concluded no uptake difference between culture-positive and -negative groups; however, its small sample size (eight positive, seven negative cases) and inclusion of diverse infection types (e.g., Brodie’s abscess, post-surgical osteomyelitis, vertebral osteomyelitis, clavicular osteomyelitis, recurrent infections) rather than standardized lower extremity osteomyelitis represent limitations compared to the present study.

Compared to ^18^F-FDG PET/CT, which has also shown promising diagnostic performance in osteomyelitis, bone SPECT/CT offers several advantages. First, the radiation dose from bone SPECT/CT using ^99m^Tc labeled agents (e.g., MDP, DPD) is generally lower than that of FDG PET/CT, making it more suitable for patients requiring serial follow-up or those with comorbidities sensitive to radiation exposure [34]. According to previous studies, the average radiation dose from FDG PET/CT was measured to be 12.2 mSv [35], while the average radiation dose from SPECT/CT was 7.7 mSv [36], although actual doses may vary considerably depending on the specific protocol or region. Second, bone SPECT/CT is more accessible in most healthcare settings, with significantly lower costs and wider availability compared to PET/CT [23,37]. In Korea, when reimbursed under the National Health Insurance Service, the cost of SPECT is approximately half that of PET, with a price difference exceeding USD 200. A previous study by Hlatky et al. reported that PET costs were 22% higher than SPECT in patients undergoing evaluation for suspected coronary artery disease in the United States [38]. According to the National Health Insurance Service database, there are 310 SPECT scanners and 170 PET scanners nationwide in Korea, which indicates a wider availability of SPECT [39]. In Japan, the national health insurance system does not cover FDG PET/CT, but only covers ^67^Ga scintigraphy for lower limb osteomyelitis [26]. These figures suggest that SPECT is generally more cost-effective and more widely available than PET, although these advantages may vary between countries. Given these advantages, bone SPECT/CT may be a more practical and equally informative tool for guiding surgical decision-making in osteomyelitis, particularly in settings with limited access to PET imaging.

The LBR cut-off identified in this study demonstrated strong predictive performance and may serve as a practical reference in clinical decision-making. In cases where the LBR exceeds the proposed threshold, proceeding with standard intraoperative cultures may be sufficient given the higher likelihood of pathogen detection. Conversely, when the LBR is below the threshold but clinical suspicion remains high, extended microbiologic work-up—such as prolonged incubation, anaerobic or fungal cultures, or molecular diagnostics—may be considered to minimize the risk of false-negative results.

This study has several limitations. First, it was a retrospective single-center study, which may limit the external validity of the results, as laboratory protocols and surgical decision-making processes can vary among institutions. For more reliable conclusions, a prospective multicenter design involving patients with suspected osteomyelitis undergoing ^99m^Tc DPD bone SPECT/CT prior to surgery would be preferable. Second, only surgically treated patients were included, introducing a potential selection bias that may overrepresent more severe or advanced cases of osteomyelitis. Third, the exact administered dose of radiotracer was not documented. As such, quantitative measures like SUV_max_ or SUV_mean_ could not be obtained. However, this also helps normalize the measurement and reduce potential error due to variable injection quality or patient-specific tracer kinetics. Fourth, propensity score matching was performed on the exact number of days of antibiotic use within four weeks prior to surgery to reduce bias from exposure length. Nevertheless, potential residual confounding from antibiotic type and cumulative prior exposure cannot be entirely excluded. Fifth, culture-negative patients were followed for long-term outcomes; among the 18 patients with ≥1 year of follow-up, 4 (22.2%) experienced reactivation of osteomyelitis. This suggests both a considerable false-negative rate in intraoperative cultures and the possibility of misclassification bias, which could underestimate the diagnostic performance of LBR. Therefore, our findings should be interpreted as predictive of intraoperative culture results rather than definitive infection diagnosis. Sixth, a misclassification bias may also occur from positive bone scans in non-infectious conditions. ^99m^Tc DPD bone SPECT/CT can show high tracer uptake in instances such as recent trauma, postsurgical changes, or other causes of increased bone turnover, potentially leading to false-positive results. As such, positive uptake should always be interpreted in the context of clinical findings, laboratory results, and complementary imaging modalities. Finally, while the AUC for LBR was meaningful, it was not sufficiently high to serve as a standalone diagnostic indicator. LBR should be interpreted in conjunction with clinical signs and other diagnostic indicators, and future studies with larger sample sizes may yield higher specificity and sensitivity.

## 5. Conclusions

In patients with suspected lower limb osteomyelitis, ^99m^Tc DPD bone SPECT/CT uptake ratio analysis may be useful in predicting tissue culture results. High tracer uptake may also be observed in various other conditions and thus should be interpreted in a multidisciplinary context in conjunction with other modalities.

## Figures and Tables

**Table 1:** Demographic and clinical characteristics for culture-positive and culture-negative groups.

	Total (*n* = 46)	Culture-Positive (*n* = 28)	Culture-Negative (*n* = 18)	*p*-Value
Age (years	57.7 ± 16.9	58.3 ± 17.4	56.8 ± 16.9	0.787
Sex (% male)	31 (67.3%)	20 (71.4%)	11 (61.1%)	0.466
Preop antibiotic use (%)	25 (54.3%)	12 (42.8%)	13 (72.2%)	0.072
Preop antibiotic use within 4 weeks (days)	9.3 ± 11.8	6.7 ± 12.5	13.4 ± 10.7	0.069
Involved bone				0.018 ^1^
Femur	8	7	1	
Tibia	33	16	17	
Calcaneus	5	5	0	
WBC (×10^3^/μL)	6.83 ± 1.59	6.96 ± 1.26	6.63 ± 1.79	0.473
ANC (×10^3^/μL)	4.23 ± 1.44	4.45 ± 1.09	3.89 ± 1.60	0.166
ESR (mm/hr)	40.2 ± 33.2	41.6 ± 34.3	38.1 ± 33.1	0.728
CRP (mg/dL)	1.61 ± 2.68	1.83 ± 2.26	1.28 ± 2.94	0.481
LBR	11.5 ± 7.8	14.5 ± 4.5	6.8 ± 8.0	<0.001

^1^ *p*-value based on distribution of involved bones across groups (Fisher’s exact test).

## Data Availability

Data to support the findings of this study are available upon reasonable request.

## Abbreviations

The following abbreviations are used in this manuscript:

SPECT/CT	Single photon emission computed tomography/computed tomography
LBR	Lesion-to-Background Ratio
